# Breaking the Pain Cycle: Hydroflotation With Promethazine and Dexamethasone for Post-laparoscopic Shoulder Pain

**DOI:** 10.7759/cureus.98544

**Published:** 2025-12-05

**Authors:** Abubakar Elmagamr, Mouhammad Halabi, Kayanne Khoury, Abdulrahman Alomar, Khadija Hayyan, Engy Bishara, Hatem Mousa

**Affiliations:** 1 School of Medicine, Royal College of Surgeons in Ireland - Bahrain, Busaiteen, BHR; 2 Surgery, Royal College of Surgeons in Ireland - Bahrain, Busaiteen, BHR; 3 General Surgery, American Hospital Dubai, Dubai, ARE

**Keywords:** hydroflotation, laparoscopic surgery, minimally invasive surgery, post-laparoscopic, shoulder pain

## Abstract

Background: Minimally invasive surgery (MIS) has become the gold standard in modern surgical practice due to its numerous advantages, including reduced intraoperative blood loss, shorter hospital stays, and faster recovery times. However, MIS is not without limitations, and one common postoperative complication is post-laparoscopic shoulder pain (PLSP). This study aims to evaluate the effectiveness of a novel hydroflotation technique utilizing Ringer’s solution combined with promethazine and dexamethasone to reduce the incidence and severity of PLSP, thereby addressing a key barrier to enhanced postoperative recovery.

Methods: This study investigates a novel hydroflotation technique in which Ringer’s solution, promethazine, and dexamethasone are instilled into the abdominal cavity following laparoscopic surgery. The combination aims to mitigate diaphragmatic irritation and inflammation caused by residual CO_2_. Patients were monitored postoperatively for the incidence and severity of PLSP, and outcomes were recorded and analyzed.

Results: Patients who underwent the hydroflotation technique experienced a notable reduction in the severity of PLSP compared to conventional postoperative management. The combination of Ringer’s solution with promethazine and dexamethasone demonstrated potential in alleviating diaphragmatic irritation and inflammation, contributing to improved postoperative recovery.

Conclusion: The hydroflotation technique shows promise as a novel intervention for reducing PLSP following MIS. By addressing a significant postoperative complication, this technique has the potential to enhance recovery outcomes and patient satisfaction. Further studies with larger cohorts are needed to validate these findings and assess the broader applicability of this method.

## Introduction

Minimally invasive surgery (MIS) has become the preferred approach for numerous procedures, with many routine operations transitioning to this technique [1-4]. Its widespread adoption is supported by well-documented advantages, including reduced intraoperative blood loss, shorter hospital stays, improved cosmetic outcomes, faster recovery, and fewer postoperative complications [3-5]. Despite these benefits, MIS is not without drawbacks. One of the most prevalent and discomforting postoperative issues is post-laparoscopic shoulder pain (PLSP), reported in approximately 35-80% of patients [5]. This pain is primarily attributed to diaphragmatic irritation caused by residual carbon dioxide (CO_2_) within the peritoneal cavity, which stimulates the phrenic nerve through both mechanical pressure and chemical irritation from carbonic acid formation [6].

Although PLSP is self-limiting, it can delay mobilization, prolong analgesic use, and diminish the overall recovery experience. Efforts to mitigate this pain have focused on minimizing residual CO_2_ and reducing diaphragmatic irritation through strategies such as low-pressure pneumoperitoneum, warmed and humidified CO_2_, pulmonary recruitment maneuvers, and intraperitoneal gas drainage. However, outcomes across studies have been inconsistent [4].

Hydroflotation, which infuses fluid into the abdominal cavity at the end of surgery, has emerged as a promising method to displace retained CO_2_ and reduce peritoneal irritation. Building on this concept, the current study evaluates a modified hydroflotation technique combining Ringer’s solution with promethazine and dexamethasone, which together provide mechanical separation, antihistaminic effects, and anti-inflammatory benefits that may collectively mitigate phrenic nerve irritation.

This study assesses the impact of this hydroflotation approach on the incidence and severity of PLSP in patients undergoing minimally invasive gynecologic surgery for benign conditions. The study aims to provide early evidence regarding its clinical effectiveness, safety, and feasibility, thereby contributing to ongoing efforts to optimize postoperative pain management in MIS. This report adheres to the Preferred Reporting Of Case Series in Surgery (PROCESS) 2023 guidelines for surgical studies [7].

## Materials and methods

This prospective, non-randomized pilot study was conducted at the American Hospital Dubai, a tertiary care referral center, between February 2024 and July 2024.

Study population and sampling

Patients were eligible if they were ≥18 years of age and underwent laparoscopic or robotic-assisted surgery for a benign gynecologic condition.

Exclusion criteria included procedures performed for malignant indications, incomplete medical records, allergy or contraindication to promethazine or dexamethasone, severe cardiopulmonary comorbidity precluding standard pneumoperitoneum, and refusal to provide informed consent.

Malignant cases were excluded because oncologic procedures typically require longer operative times, more extensive tissue manipulation, and postoperative adjuvant therapy, all of which could independently affect pain perception and recovery. Patients were recruited consecutively during the study period to minimize selection bias.

Procedure details

All procedures were performed in the Trendelenburg position at an angle between 25° and 30°, using four 8-mm ports with placement tailored to patient anatomy and surgical requirements. The abdominal cavity was insufflated with CO_2_ to 15 mmHg at a maximum flow rate of 2 L/min.

At the end of each operation, CO_2_ was passively evacuated while gentle abdominal pressure was applied. While patients remained in Trendelenburg, 2 L of Ringer’s solution containing 25 mg promethazine and 4 mg dexamethasone was instilled bilaterally into the upper abdomen. The solution was left in situ and not aspirated.

The choice of 2 L was based on prior hydroflotation studies [8,9], which demonstrated effective CO_2_ displacement and diaphragmatic protection without compromising hemodynamics. Continuous intraoperative monitoring confirmed the safety of this volume in our cohort. The control (“non-hydroflotation”) group underwent identical procedures and postoperative protocols, excluding the final instillation step.

Outcomes and definitions

The primary outcome was the severity of postoperative shoulder pain, measured on postoperative day 1 using the Numeric Rating Scale (NRS) from 0 (no pain) to 10 (worst imaginable pain) [10]. Secondary outcomes included operative complications, estimated blood loss, hospital stay duration, and the requirement for rescue analgesia beyond the standardized regimen.

Perioperative management

All patients received the same standardized postoperative analgesia: intravenous parecoxib 40 mg every six hours for three doses and paracetamol 1000 mg every six hours for three doses, followed by as-needed dosing thereafter. No additional intraoperative analgesics were administered.

Statistical analysis

Baseline demographic and perioperative characteristics were summarized as mean ± standard deviation for continuous variables and as frequencies and percentages for categorical variables. Student’s t-test was used for continuous variables, and chi-square or Fisher’s exact test for categorical variables, as appropriate. A two-tailed p-value <0.05 was considered statistically significant.

Operative time was not included in the statistical analysis because case duration is influenced by multiple factors, such as adhesiolysis, surgical complexity, and operator technique, that can confound pain outcomes without directly reflecting diaphragmatic CO_2_ exposure. Moreover, operative durations showed limited variability between groups, reducing the ability to detect meaningful differences. Future larger studies should stratify by operative complexity to explore this relationship.

Ethical approval

The study protocol was reviewed and approved by the Institutional Review Board (IRB) of American Hospital Dubai (approval number: AHD-IRB-154). All procedures adhered to the ethical principles of the Declaration of Helsinki. Written informed consent was obtained from all participants prior to inclusion.

## Results

A total of 55 patients were included in this study, with 27 patients in the hydroflotation group and 28 in the control group. The two groups were comparable in terms of demographics, including age, body mass index (BMI), and medical history. Baseline characteristics are summarized in Table 1. The most common comorbidity in both groups was anemia, and no significant differences were observed in preoperative medications or American Society of Anesthesiologists (ASA) classification.

**Table 1:** Baseline Demographics and Preoperative Characteristics of Patients Undergoing Minimally Invasive Surgery With and Without Hydroflotation COPD: chronic obstructive pulmonary disease; ASA: American Society of Anesthesiologists

Variable	Hydroflotation (n=27)	No Hydroflotation (n=28)	Test Statistic	P-value
Age (years)	38.22 ± 5.8	39.89 ± 8.58	t = 0.64	0.526
BMI (kg/m^2^)	26.18 ± 4.22	27.21 ± 6.71	t = 0.53	0.6
Smoking history - Prior smoker	12 (44%)	18 (64%)	χ^2^ = 1.46	0.228
Smoking history - Current smoker	15 (56%)	10 (36%)	χ^2^ = 1.46	0.228
Hypertension	1 (3.7%)	1 (3.6%)	N/A	N/A
Hypothyroidism	6 (22%)	1 (3.6%)	χ^2^ = 2.77	0.095
Diabetes	1 (3.7%)	0	χ^2^ = 0.00	0.985
COPD	1 (3.7%)	0	χ^2^ = 0.00	0.985
Coronary artery disease	0	0	N/A	N/A
Congestive heart failure	0	0	N/A	N/A
ASA ≤3	25 (93%)	28 (100%)	χ^2^ = 1.40	0.236
ASA ≥4	2 (7.4%)	0	N/A	N/A
Prior surgery	2 (7.4%)	1 (3.6%)	χ^2^ = 0.00	0.974
Prior gynecologic surgery	9 (33%)	8 (29%)	χ^2^ = 0.01	0.928
Preoperative aspirin	0	0	N/A	N/A
Preoperative statin	10 (37%)	4 (14%)	χ^2^ = 2.65	0.104
Preoperative beta-blocker	0	0	N/A	N/A

Operative findings are detailed in Table 2. Among the 55 procedures, 38 (69%) were conventional laparoscopic and 17 (31%) were robotic-assisted surgeries, with a comparable distribution between groups (p = 0.58). No intraoperative complications or conversions to open surgery were observed. The mean estimated blood loss was similar between the two groups (190.5 ± 47.0 mL vs. 190.6 ± 45.6 mL; p = 0.991). In the hydroflotation group, two patients (7.4%) had an estimated blood loss greater than 100 mL, compared with two patients (10.7%) in the control group.

**Table 2:** Intraoperative Findings and Operative Metrics Comparing Hydroflotation and Control Groups

Variable	Hydroflotation (n=27)	No Hydroflotation (n=28)	Test Statistic	P-value
Hysterectomy	10 (37%)	16 (57%)	χ^2^ = 3.62	0.312
Myomectomy	10 (37%)	11 (39%)	χ^2^ = 3.62	0.312
Cystectomy	5 (19%)	2 (7%)	χ^2^ = 3.62	0.312
Adenomyomectomy	1 (3.7%)	0	χ^2^ = 3.62	0.312
Conversion to open surgery	0	0	N/A	N/A
Intraoperative complications	0	0	N/A	N/A
Estimated blood loss (mL)	190.50 ± 47.02	190.64 ± 45.60	t = 0.01	0.991

Postoperative outcomes are summarized in Table 3. The shoulder pain score on postoperative day 1 was significantly lower in the hydroflotation group compared with the control group (p < 0.001). Twenty-three patients (85%) in the hydroflotation group reported mild or minimal pain (NRS < 5), whereas 21 patients (75%) in the control group reported moderate to severe pain (NRS ≥ 5). The length of hospital stay was comparable between groups (p = 0.194), with most patients discharged on the same day or within 24 hours postoperatively.

**Table 3:** Postoperative Shoulder Pain and Hospital Stay Outcomes in Patients With and Without Hydroflotation POD1: postoperative day 1; NRS: Numeric Rating Scale

Variable	Hydroflotation (n=27)	No Hydroflotation (n=28)	Test Statistic	P-value
Hospital stay 0-1 days	20 (74%)	15 (54%)	χ^2^ = 1.68	0.194
Hospital stay >1 day	7 (26%)	13 (46%)	χ^2^ = 1.68	0.194
Pain score POD1 - NRS <5	23 (85%)	7 (25%)	χ^2^ = 19.82	<0.001
Pain score POD1 - NRS ≥5	4 (15%)	21 (75%)	χ^2^ = 19.82	<0.001

In addition to standardized postoperative analgesia, the need for rescue morphine (2 mg IV) was significantly lower among patients who underwent the hydroflotation technique, with only three patients (11%) requiring additional doses compared with 12 patients (43%) in the control group (χ^2^ = 7.21, p = 0.007).

Throughout the study, no major postoperative complications were recorded. One patient in the hydroflotation group developed a transient postoperative fever on the first postoperative day that prolonged the hospital stay to three days; all investigations were negative, and the patient recovered uneventfully following a single dose of intravenous antibiotics.

## Discussion

MIS has transformed surgical care by offering faster recovery, reduced postoperative pain, and fewer complications compared with traditional open procedures. However, PLSP remains a common and often under-addressed complication, affecting 35-80% of patients [5]. In this study, we evaluated a novel and simple technique involving hydroflotation with Ringer’s solution combined with promethazine and dexamethasone to reduce PLSP. Our findings demonstrate a significant reduction in pain severity and in the need for additional analgesia among patients who received hydroflotation, supporting the feasibility and potential benefit of this approach in enhancing postoperative comfort and accelerating recovery after MIS.

Although the exact mechanism of PLSP is multifactorial, diaphragmatic irritation caused by retained CO_2_ is thought to be the principal trigger. Various methods have been explored to reduce this irritation, including low-pressure pneumoperitoneum, warmed and humidified CO_2_, intraperitoneal gas evacuation, local anesthetic instillation, and pulmonary recruitment maneuvers [4]. However, their efficacy and cost-benefit ratio remain inconsistent. Promethazine, an antihistamine, may mitigate neural irritation and neuropathic discomfort by stabilizing histaminergic pathways [11], whereas dexamethasone offers potent anti-inflammatory effects that can limit local irritation and inflammatory response after surgery. The combined mechanical (hydroflotation) and pharmacologic (anti-inflammatory and antihistaminic) effects may act synergistically to minimize phrenic nerve stimulation and reduce PLSP intensity.

Our results indicate that, irrespective of BMI, prior surgical history, or comorbidities, hydroflotation with Ringer’s solution, promethazine, and dexamethasone led to significantly less PLSP compared with conventional postoperative management. Furthermore, the need for rescue opioid analgesia was substantially lower in the hydroflotation group, suggesting both a clinical and economic advantage. From a healthcare-economics perspective, reducing postoperative pain can translate into shorter recovery times, decreased opioid consumption, and faster discharge - all of which contribute to improved patient satisfaction and reduced overall resource utilization. These benefits are particularly relevant in high-volume centers where even modest reductions in postoperative pain can have measurable cost implications.

The current literature presents mixed results regarding the use of hydroflotation for PLSP prevention. Tsai et al. demonstrated that intraperitoneal saline instillation significantly reduced PLSP severity and incidence [8,12], while Suginami et al. similarly reported that fluid instillation effectively displaces CO_2_ and prevents diaphragmatic irritation [9]. Conversely, van Dijk et al. found no significant benefit from saline hydroflotation when combined with pulmonary recruitment [13]. Dexamethasone alone has shown promise: Asgari et al. found that 16 mg intraperitoneal dexamethasone significantly reduced PLSP intensity (p < 0.001) [14]. Meanwhile, Zhao et al. reported that intravenous lidocaine reduced abdominal discomfort but not shoulder pain following gynecologic laparoscopy [15]. Beyond pharmacologic interventions, several noninvasive modalities, including transcutaneous electrical nerve stimulation (TENS), acupuncture, and massage therapy, are being investigated for their potential to alleviate PLSP [16]. However, recent evidence, including the trials by Olguín-Ortega et al. and Temtanakitpaisan et al., suggests that mechanical or physiotherapeutic interventions alone may not fully address the complex pathophysiology underlying PLSP [17,18].

Procedure duration also plays a critical role in PLSP development. Longer operative times can increase CO_2_ exposure and prolong Trendelenburg positioning, both of which enhance diaphragmatic irritation and inflammatory activation. While operative duration was recorded, it was not statistically analyzed in this pilot due to limited variation across cases, and the multifactorial nature of the variable factors, such as adhesiolysis or surgical complexity, may independently influence operative time without directly reflecting CO_2_ exposure. Future studies with larger, stratified samples should evaluate operative duration more systematically to delineate its relationship to PLSP.

Several limitations of this study warrant discussion. The study design inherently limits the ability to establish causality, and the absence of randomization or blinding may have introduced selection and reporting bias. Pain scores were collected in the presence of medical staff, which may have influenced patient responses. The small sample size and single-center setting limit the generalizability of the findings, although consistency across patient characteristics strengthens internal validity. Furthermore, the intervention combines multiple active components - Ringer’s solution, promethazine, and dexamethasone - making it difficult to isolate the individual contribution of each agent. Despite these limitations, the study demonstrates that the technique is safe, easily reproducible, and practical for integration into standard surgical workflows.

Future research should focus on conducting multicenter randomized controlled trials to validate these preliminary findings, optimize drug dosing, and assess the applicability of this technique across different laparoscopic specialties. Additionally, evaluating outcomes such as postoperative opioid consumption, patient satisfaction, and early ambulation could further define the broader clinical and economic benefits of hydroflotation.

## Conclusions

Hydroflotation with Ringer’s solution, promethazine, and dexamethasone significantly reduced PLSP and the need for additional analgesia in this pilot study. The technique is simple, safe, and easily incorporated into existing surgical workflows. By improving patient comfort and recovery, hydroflotation shows promise as a low-cost adjunct for postoperative pain management. Larger multicenter studies are needed to confirm these findings and refine dosing protocols.
